# The complete chloroplast genome of *Paris polyphylla* var. *chinensis*, an endemic medicinal herb in China

**DOI:** 10.1080/23802359.2019.1687351

**Published:** 2019-11-08

**Authors:** Xujun Wang, Cuiying Peng, Junsheng Liang, Qidong Liang, Caili Xu, Wei Guo

**Affiliations:** a Hunan Academy of Forestry, Changsha, P. R. China;; b College of Forestry, Central South University of Forestry and Technology, Changsha, P. R. China;; c College of Agronomy, Hunan Agricultural University, Changsha, P. R. China;; d Taishan Academy of Forestry Sciences, Taian, P. R. China

**Keywords:** *Paris polyphylla* var. *chinensis*, chloroplast genome, Illumina sequencing

## Abstract

*Paris polyphylla* var. *chinensis* is a species of flowering herb of the family Liliaceae and widely distributed in 12 provinces in China. It has been used in Chinese traditional medicine for centuries. The chloroplast (cp) genome of *P. polyphylla* var. *chinensis*, sequenced based on next-generation platform (NEOSAT), is 164,429 bp in size. The cp genome encodes 133 genes, including eight rRNA genes, 87 protein-coding genes (PCGs), and 38 tRNA genes. Phylogenetic relationship analysis based on complete cp genome sequences exhibited that *P. polyphylla* var. *chinensis* was most related to *Daiswa forrestii*.

*Paris polyphylla* var. *chinensis*, an endemic to China, is a species of flowering herb of the family Liliaceae and widely distributed in 12 provinces in China (Wang 1978). In traditional Chinese medicine, its dried rhizomes, also well known as Rhizoma Paridis (Chonglou), have been used for the treatment of hemostasis, sore throat, parotitis, furuncle, carbuncle, snake bite, and convulsion. Most previous studies have concentrated mainly on its geographical distribution and chemical compounds (Kang et al. 2017; Cunningham et al. 2018). Steroidal saponins obtained from its rhizomes displayed cytotoxic activity against various tumor cells (Zhang et al. 2015; Liu et al. 2016; Xie et al. 2017). In recent years, its natural population decreased drastically because of deforestation. Hence, the genomic and genetic knowledge is urgently needed to promote a new strategy to conserve and make good use of the germplasm resources of *P. polyphylla* var. *chinensis*. Therefore, in this paper, its cp genome sequences were reconstructed.

The voucher specimen (accession no. EGD_1241_HCL_TxdCS) was sampled from a *P. polyphylla* var. *chinensis* plant at Dujiachong experimental forest located in Yuhua District, Changsha, Hunan, China (28°06′40″N, 113°01′30″E). It was frozen in a dry ice-ethanol bath and deposited in a deep freezer at the herbarium of Hunan Academy of Forestry. An Illumina Hiseq 2500 system was employed to carry out paired-end sequencing. After trimming, around 7.2 Gb clean data were assembled against the online published cp genome of *P. rugosa* (GenBank no. KY247142; Song et al. 2017) using bowtie2 (Langmead and Salzberg 2012). Filtered reads were pooled and used for *de novo* assembly. The scaffolds were obtained by SSPACE v2.0 (Bankevich et al. 2012) and supplemented by Gapfiller v2.1.1 (Boetzer and Pirovano 2012). The annotation was achieved using ARAGORN v1.2.38 (Laslett and Canback 2004), HMMER v3.1b2 (Finn et al. 2011) and BLAST searches, then corrected manually.

The complete cp genome of *P. polyphylla* var. *chinensis* (GenBank no. MN528722) is a circular DNA molecule with 164,429 bp in size having 36.94% of total GC content. It contains two inverted repeat regions (IRs, 33,365 bp each) with GC content 39.62%, a small single-copy region (SSC, 12,968 bp) with GC content 32.16% and a large single-copy region (LSC, 84,731 bp) with GC content 35.56%. There are a total of 133 genes annotated in the cp genome, comprising eight rRNA genes, 87 PCGs and 38 tRNA genes. Eleven tRNA genes, nine PCGs, and four rRNA genes were duplicated in the IRs. There are six intron-bearing PCGs. Two of them (*ycf3* and *clpP*) bear two introns each, while four of them (*atpF*, *rpl2*, *rpoC1*, *ndhA*), bear one intron each.

For phylogenetic maximum likelihood (ML) analysis, multiple alignment was completed by MAFFT v7.2 with cp genomes downloaded from Genbank with default parameters (Katoh and Standley 2013). The ML tree, inferred using mega v10.0.4 (Kumar et al. 2018) with 1000 bootstraps, presented that *P. polyphylla* var. *chinensis* was most related to *Daiswa forrestii* (Figure 1).

**Figure 1.**
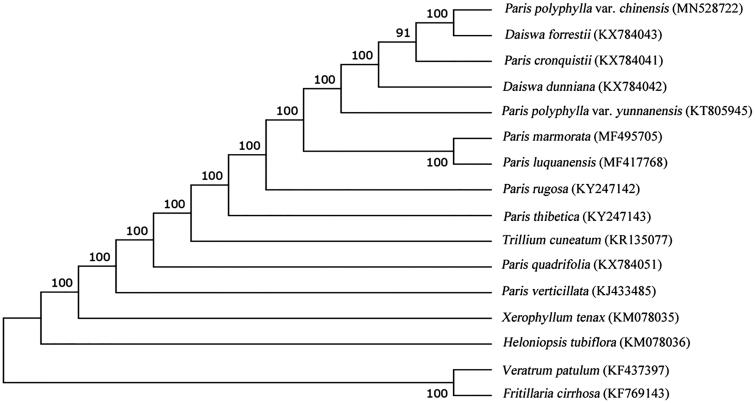
Phylogenetic tree based on 16 complete cp genome sequences. The bootstrap support values are shown next to the branches.
